# Changes in Nitric Oxide Releases of the Contralateral Acupoint during and after Laser Acupuncture at Bilateral Same-Name Acupoints in Human

**DOI:** 10.1155/2017/5763458

**Published:** 2017-11-20

**Authors:** Wan-Ling Jiang, Hua-Jiang Wei, Zhou-Yi Guo, Yi-Rong Ni, Hong-Qin Yang, Shu-Sen Xie

**Affiliations:** ^1^MOE Key Laboratory of Laser Life Science & SATCM Third Grade Laboratory of Chinese Medicine and Photonics Technology, College of Biophotonics, South China Normal University, Guangzhou 510631, China; ^2^Key Laboratory of Optoelectronic Science and Technology for Medicine of Ministry of Education of China, Fujian Normal University, Fuzhou, Fujian 350007, China

## Abstract

**Objective:**

The purpose of the study was to examine the effects of laser acupuncture (LA) at right Neiguan (RPC6)/left Neiguan (LPC6) acupoints on the releases of nitric oxide (NO) in the treated and contralateral/nontreated PC6, compared to the nonacupoint control area.

**Methods:**

24 mW LA at RPC6, LPC6, and nonacupoint in 22 healthy subjects for 40 min: sterilized dialysis tube was taped to the nontreated PC6/nonacupoint during the treatment and immediately taped to the treated and nontreated PC6/nonacupoint after LA removal. NO-scavenging compound was injected into the tube for 40 min to absorb the molecular which was tested by spectrophotometry in a blinded fashion.

**Results:**

LA-induced NO releases over PC6 acupoints for the nontreated and treated sides all significantly increased after LA removal, but for the nontreated acupoints they did not change during LA stimulation. LA at RPC6 induced the more release of the NO at contralateral side than stimulating LPC6, but not on nonacupoints. The results suggest that LA-induced NO release over contralateral acupoint and NO release resulting from the lateralized specificity all are different and specific to the acupoint within different time course.

**Conclusions:**

LA-evoked NO release over acupoints could improve the neurogenic, endothelial activity of the vessel wall to further facilitate microcirculation.

## 1. Introduction

Acupuncture as an important component of Traditional Chinese Medicine (TCM) serves as an extensive treatment approach of diseases in clinical practice, which dates back about 2000 years [1]. According to the TCM theory, acupoints are distributed along the meridian pathways system; meridian systems are believed to deal with pathological changes and physiological regulation of the human body as physical pathway systems. Based on the principles of* Huang Di Nei Jing Su Wen* [2], the clinical effects of acupuncture might be closely connected with the appropriate acupoints selection during the treatment. For example, acupuncture or laser acupuncture (LA) stimulation of the Neiguan acupoint (PC6), located on the pericardium (PC) Hand-JueYin meridian, is recommended for the treatments of nausea, cardiac and gastric pain, or stress management [3].

Additionally, there are some evidences concerning the specificity of acupoints that seem to be confirmed; many researchers pointed out that LA and electroacupuncture significantly increased nitric oxide (NO) release over acupoint whereas the NO release over nonacupoint only changed slightly after the same treatment [4–6]. Needling at acupoint enhanced the blood perfusion of the acupoint, but not at nearby nonacupoint [7]. Acupuncture increased the blood NO content of the acupoints [8]. However, the biochemical transmission mechanisms of acupuncture signal and specificity of acupoints are still unknown.

Actually, there were different methods of acupuncture treatment. Compared to the traditional invasive acupuncture, LA as an increasingly common clinical method has been used in primary care and scientific research; it is capable of relieving pain [9] and not injuring the patient, which can not only simulate traditional acupuncture but reduce the stress effect caused by the mental needle [10]. Herein, we select laser needle as research tool and explore LA-induced biochemical signal molecule: NO changes for the PC6 beneficial effects.

Previous studies have demonstrated that NO levels over acupoints were higher than those of the nonacupoints [4, 11, 12]. NO as an important messenger molecule wields a significant influence on modulating the vasodilatation and vessel smooth muscle relaxation [13]; the vascular endothelial cells are capable of synthesizing NO as the primary cells in the body. It is well-known that NO could be rapidly oxidized into nitrite (NO_2_^−^) and nitrate (NO_3_^−^) because of its chemical lability; these two stable metabolites serve as the very adequate indicators to measure the amount of NO from the tissue and the changes in NO activity [14, 15].

It is reported that LA at right acupoint induced the blood perfusion increase of the contralateral acupoint, although the LA was ceased, the blood perfusion is still significantly increasing, but there are no significantly changes in nonacupoint [16]. Electroacupuncture-induced NO synthesis could increase hepatic blood perfusion via vasodilation in liver tissue [17]. Our recent finding showed that LA-evoked releases of NO over acupoints are significantly enhanced compared to nonacupoint [4]; in this study, the purpose is to investigate the changes in NO levels of the PC6 for both nontreated and treated sides during and after LA treatments and to investigate whether the NO releases of bilateral PC6 with the same name are different during and after the same LA treatments and whether these phenomena are specific to acupoints.

## 2. Methods

*Registration.* The study was approved by the Chinese Clinical Trial Registration, with the registration number: ChiCTR-BOC-17011442 (AMCTR-IOO-17000061); the registration date is 2017/5/21.

### 2.1. Subjects and Research Program

22 normotensive, nonsmokers, healthy volunteers who participated in this study (10 males, 12 females) (mean ± standard error (SE), age: 25.4 ± 3.4 years, body mass: 62.2 ± 10.2 kg, height: 171.5 ± 9.5 cm) were recruited from South China Normal University and fully informed of the purpose of this study as well as the procedures to be utilized. They provided the written informed consent and obtained detailed oral instructions of this study. Our research was a randomized, single-center, cross-over, and single-blind fashion and conducted ethically according to international standards and the requirement of the journal. The study was approved by the Laboratory of Photonic Chinese Medicine, the Key Laboratory Institute of the education Ministry of Laser Life Science, South China Normal University, and abided by their requirements for human experimentation. All participants did not suffer from any history of cardiovascular disease, major surgery, vascular disorders, allergic disease, prescribed medication, infectious diseases, or dermatological problems in the past year. Women participants would be excluded from the study if they were in their menstrual period or pregnant. Due to the fact that the movement of the subject's arm and the external NO metabolites (NO_*x*_^−^) from diet could influence the experimental measurements, each subject was instructed to stop fasting or drinking before more than 2 hours and then sit comfortably on the chair, and their arms were fixed using cellophane tape. Experiments were performed at the same time of each day and in restful, calm conditions with the room temperature at 24–26°C the relative humidity maintained at 45%  ± 15% to eliminate the influence of different surroundings.

### 2.2. Identification of Acupoints and LA Stimulation

**Figure 1:**
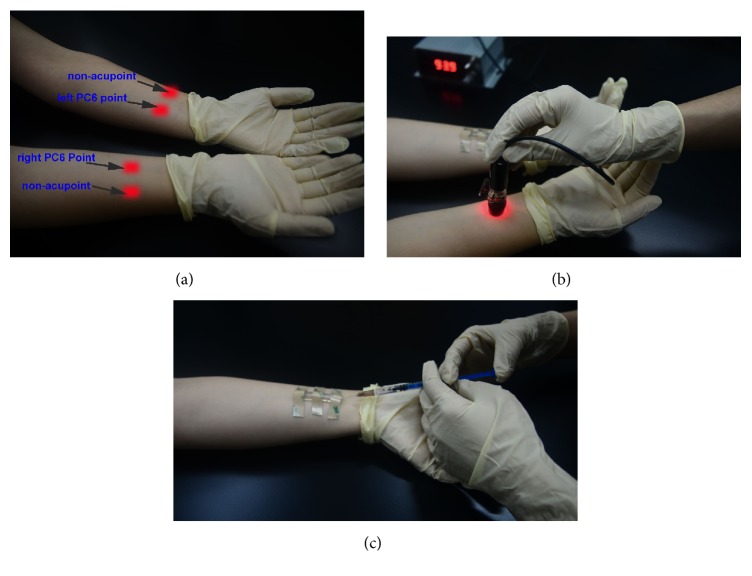
*Schematic illustration of the biocapture device and LA at relevant points:* (a) at right PC6 acupoint (RPC6) and right nonacupoint (Rnon-acupoint) (bottom); at left PC6 acupoint (LPC6) and left nonacupoint (Lnon-acupoint) (top). (b) LA at RPC6 for 40 min and NO was collected from the LPC6 acupoint. (c) 0.3 ml PTIO solution (200 *μ*M) was injected into the sterilized dialysis tube (0.5 × 5 cm) and directly contacted with LPC6 for 40 min. The experiment was performed at the same time of each day and in restful, calm conditions with the room temperature at 24–26°C, the relative humidity maintained at 45%  ± 15%.

The locations of PC6 acupoints on the human forearm were determined by an acupuncturist. LA was respectively applied to right PC6 (RPC6), left PC6 (LPC6) along the PC meridian and its nearby nonacupoint ~10 mm apart with six-time repetition (Figure 1(a)). Considering that the PC6 acupoints are easily identified and serve as the effective and representative treatment for the pain [3], the NO capture tube is sufficiently placed on the skin surface, herein, the PC6 acupoints were chosen to test in this study.

A 658 nm prototype fiberoptic infrared laser with 49 mW capacity (Nanjing to the Laser Technology Co., Ltd., ED658-100) was used in the study and the laser parameters are applicable to the clinical study [10]. The projected body parts were fully swabbed using sterile distilled water and then dried prior to LA treatment. A stably held laser which its output power was designed for 24 mW was applied to the projected acupoints for 40 min, the laser was preheated for 10 min, and the preheat time would be increased if the subject did not experience a sensation of “de qi” [3] before LA treatment. All subjects should not have the experience of LA treatment prior to this experiment. LA stimulation was applied to PC6 on the nontreated/contralateral arm as PC6 dialysis (Figure 1(b)), after removal of the LA, on the treated arm as PC6 dialysis, and on the contralateral arm as PC6 dialysis; the same treated way was performed at nonacupoint. Each measured point following LA stimulation for one subject was treated once a day, the interval of 2 days was appropriate for treating the other projected acupoints, and each measurement was executed at the same time of each day.

### 2.3. NO Biocapture Process

Smart and special method which was developed by Ma et al. [11, 12] was used to examine NO generation at the treated area (Figure 1). They adopted the chemiluminescence method to measure the NO amounts, we have measured them by spectrophotometry in the study. A molded semicircular silicone-plastic tube (0.5 × 5 cm) (Texas Jin Guanghua Glass Co., Ltd., China) was adhered to the surface of the projected acupoints using a custom double-sided adhesive. The 0.3 ml 2-phenyl-4,4,5,5-tetramethylimidazoline-1-oxyl 3-oxide solution (PTIO, 200 *μ*M) (Tokyo Chemical Industry Co., Ltd., 6-15-9-Toshima, Japan) was injected into the inside of the sterilized dialysis tube and thereby can directly contact with the projected acupoints surface for 40 min to absorb NO production (Figure 1(c)). The dialysate collected from the skin surface was transferred to a new sterile tube and stored in −80°C for the measurements of NO_*x*_^−^ (NO_2_^−^, NO_3_^−^).

### 2.4. Measurement of NO Metabolites

**Figure 2:**
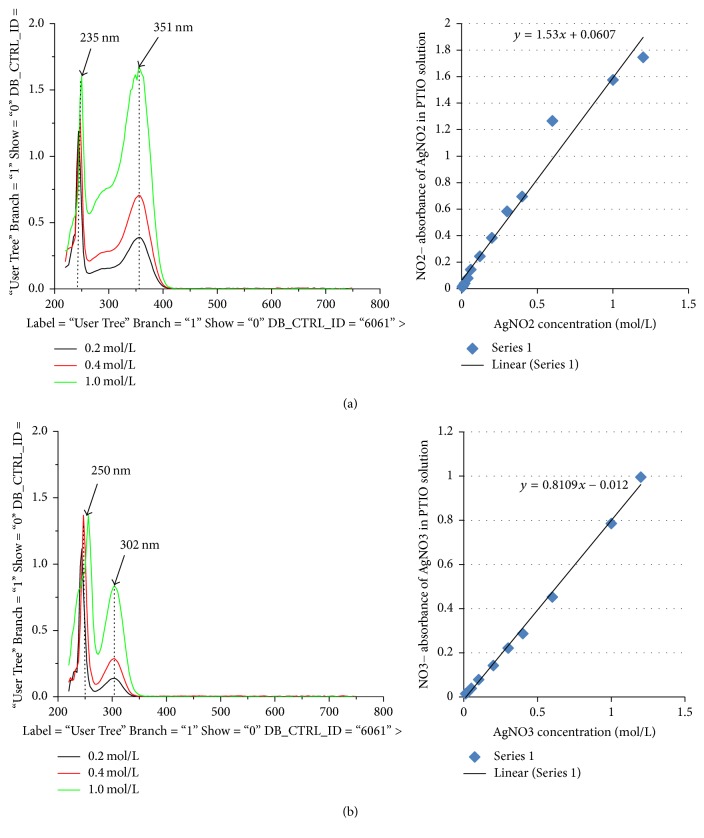
*Standard spectra of AgNO*_2_ *and AgNO*_3_ *in PTIO solutions were measured*. The standard absorption peaks of different concentrations AgNO_2_ and AgNO_3_ dissolved PTIO solution were established. The first absorption peak of AgNO_2_ solution reflects at wavelength around 235 nm and second peak reflects at around 351 nm, which fully accords with the NO_2_^−^ curves measured from our dialysate samples under the same conditions. Due to the fact that the linear relationship between the absorbance and the amount of NO_2_^−^/NO_3_^−^ can be determined from the right panels, the total amounts of NO captured by PTIO solution can be quantified according to the NO_2_^−^ absorbance in our dialysate sample.

**Figure 3:**
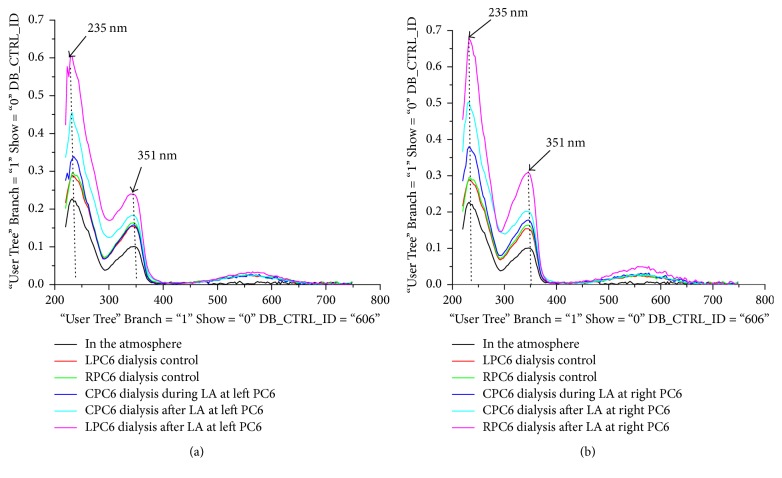
*Changes of NO release at contralateral PC6 acupoints during LA treatment and recovery period.* As described in Figure 2, due to the easy saturation of NO_2_^−^ absorbance at 235 nm, the absorbance at 351 nm was used to quantify the levels of NO which can rapidly react with PTIO solution to produce NO_2_^−^. It can be clearly shown that the NO_2_^−^ absorbance at contralateral PC6 (CPC6) did not change during LA PC6 compared with those of CPC6 control group, but significantly increased after LA removal. Additionally, the amount of NO from the air was significantly lower than that of the skin area.

There is a linear relationship between the absorbance and concentrations of nitrobenzene/protein [18, 19]; in our research, we have also demonstrated that the linear relationship between absorbance and NO_3_^−^/NO_2_^−^ concentrations at 302/351 nm peaks as shown in Figures 2(a) and 2(b) (ND-1000, NanoDrop spectrophotometer, Gene Company Limited). LA at PC6, nonacupoint for 40 minutes, produced typical three peaks of absorbance spectra in PTIO solution which was injected to special silicon tube placed on PC6 just after LA treatment (Figure 3) [4]. Based on the previous studies, the first (235 nm) and second (351 nm) peaks came from NO_*x*_^−^ [20–22]. We analyzed the standard molecules under the same conditions to confirm this speculation. We have discovered that the first peak at 235 nm and the second peak at 351 nm all came from NO_2_^−^ (Figures 2(a) and 2(b)) [4]. Additionally, the NO_2_^−^ absorbance at 235 nm was easily saturated; thus, absorbance peak at 351 nm was used to evaluate the levels of NO trapped by PTIO solution [4]. Researchers in the operation of the nitrogen oxide absorption detection, performing the assay of the absorption spectrum, dealing with the data obtained, were blinded to LA treatment.

### 2.5. Statistical Analysis

All values were reported as the means ± SE; analysis of the results was performed by using a statistical product and service solutions 16.0 software paired test. A *P* value < 0.05 is defined as a significant difference.

## 3. Results

### 3.1. Time Responses of Dialysate NO Metabolites from PC6 and Nonacupoint Area during LA Treatment and Recovery Period (after LA Removal)

We performed twenty-eight microdialysis experiments to demonstrate that these peaks were really from treated area; firstly, we placed the special biocaptured tube which was injected into PTIO solution on the skin and in the air for the same duration, then analyzed the NO_2_^−^ absorbance, and discovered that the NO_2_^−^ amount in PTIO solution collected from skin regions was obviously higher compared with that of the air (Figure 3, Table 1). This demonstrated that this method can be used to trap NO came from skin in the study, which supported our previous finding [4].

**Figure 4:**
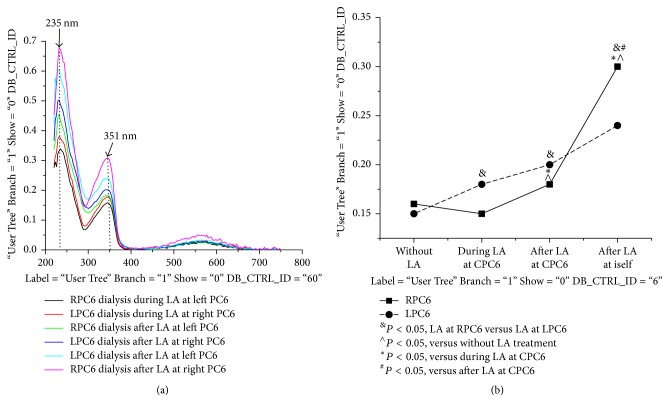
*Different releases of NO generated from bilateral same-name acupoints during and after LA at one side*. The panels show that the NO_2_^−^ absorbance of PC6 acupoints for the nontreated sides did not change during LA treatment compared to those of the PC6 without LA treatment (^∧^*P* < 0.05); the NO_2_^−^ absorbance of the PC6 for the treated and nontreated side all significantly increased after LA removal (^*∗*^*P* < 0.05), but the NO_2_^−^ absorbance of the PC6 for the treated side was obviously higher than that of the nontreated side (^#^*P* < 0.05). The NO_2_^−^ absorbance in both the right and left PC6 after LA RPC6 was obviously higher than those of stimulating LPC6 (^&^*P* < 0.05).

**Figure 5:**
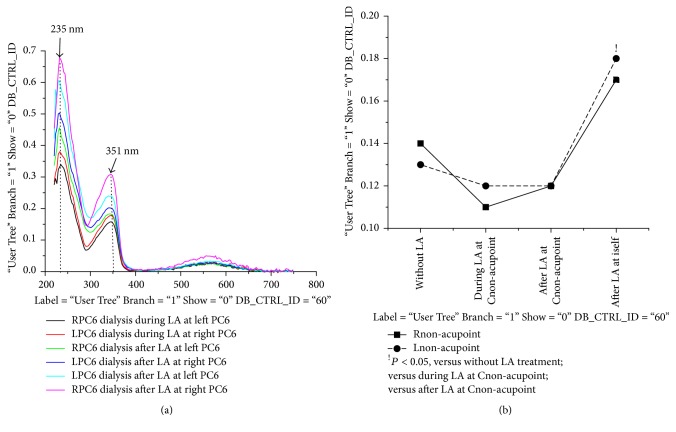
*Changes in amounts of NO came from Rnon-acupoint and Lnon-acupoint during and after LA treatment.* The panels show that the NO_2_^−^ absorbance of the nonacupoint for the nontreated sides did not change during and after LA compared with those of the nonacupoint without LA stimulation. However, LA-induced NO release over nonacupoints only for treated sides slightly increased after LA removal (^!^*P* < 0.05).

The time course changes in NO releases of the nontreated and treated PC6 during LA treatment and recovery period are shown in Table 1 and Figures 3 and 4. For the changes in LA-induced NO release, the interaction between time and side and the effects of time and side were significant. Compared to the NO baseline levels of the LPC6 and RPC6 without LA stimulation, during LA treatment, the NO_2_^−^ level of the PC6 for the nontreated side did not obviously change (*P* < 0.05). On the other hand, the NO_2_^−^ level of the PC6 for the treated and nontreated side all significantly increased after LA removal, but the NO_2_^−^ level of the PC6 for the treated side was obviously higher than that of the nontreated side (*P* < 0.05). The time course changes in NO releases of the nontreated and treated nonacupoint during LA treatment and recovery period are shown in Table 2 and Figure 5. Compared to the NO baseline levels over nonacupoint without LA stimulation, during LA treatment and recovery period, the NO_2_^−^ level of the nonacupoint for the nontreated side did not change (*P* < 0.05). However, the NO_2_^−^ level of the nonacupoint for the treated side during the recovery period suggested a moderate increase but still had statistical significance (*P* < 0.05). In addition, the increases in LA-induced NO release of the contralateral acupoint are specific to the acupoint, but not to the nonacupoint.

### 3.2. Effects of LA-Induced NO Releases in Contralateral PC6 Compared to Nonacupoint Area

Comparison of the NO release values for LPC6 and RPC6 (before the treatments) suggested no significant differences; however, the significant differences of NO release between LPC6 and RPC6 during LA treatment and recovery period are shown in Table 1 and Figure 4. LA at RPC6 has the strong amplification effects on LA-induced NO release in both RPC6 and LPC6 (*P* < 0.05). In contrast, stimulation of LPC6 only produces moderate amplification effects on LA-induced NO release in LPC6 and RPC6 (*P* < 0.05). As shown in Table 2 and Figure 5, there are no amplification effects exerted for left and right nonacupoint following the same treatments (*P* < 0.05). These results indicated that LA at lateral acupoint induced the different degree of amplification effect on NO release in contralateral side, LA at RPC6 induced the more NO release in contralateral side than stimulating LPC6, and the lateralized specificity is specific to the acupoint.

## 4. Discussion

Following the symmetrical principle of the Neijing theory [2, 23], the treatment point would usually be chosen in the right body, if someone has disease in the left side, and vice versa. Previous finding has also demonstrated the important effect of distinction between ipsilateral and contralateral acupuncture [24]. Acupuncture at one side acupoint induced the blood perfusion increase of the contralateral acupoint, which revealed the intrinsic correlation between contra- and ipsilateral acupoints [23, 25]. However, the activated mechanism of acupoints is still unknown; analysis of the specificity of acupoints after acupuncture-like stimulation is also difficult. The relationship between acupuncture and circulation is well recognized in recent studies. The change of the blood circulation for the muscle and skin is mediated by many peripheral and central factors such as reflex through the sympathetic nervous system [26, 27]. It is well-demonstrated that cutaneous vasodilation might be relevant to the activation of the NO receptor in the local microcirculation system [28]. It was found that acupuncture at ST36 acupoint elevated the expression of nitric oxide synthase (NOS) and enhanced the levels of NO in the central nervous system, skin points, or the peripheral blood [6, 29]. Electroacupuncture-evoked NO release induced the increase of hepatic blood perfusion via vasodilation in liver [17]. In our study, releases of NO from both LPC6 and RPC6 after LA RPC6 are greater than those of after LA LPC6, which may result in the possibility that stimulation on right acupoint by acupuncture caused the more forceful amplificatory effect compared to stimulation of left acupoint [23, 25]. These indicated that the acupoint has lateralized specificity.

NO with stronger activity in the tissue is a vasodilator and produced by NOS through the catalytic decomposition of L-arginine [17, 28]. Activating the endothelial NOS in the vascular endothelial cells, neuronal NOS in the brain, and nervous tissues and inducible NOS in the macrophage cells all can accelerate the synthesis and release of NO in human body. Moreover, the endothelial NOS catalyzed the synthesis of NO, which can dilate the blood vessel to mediate the normal microcirculation [30]. Anatomical studies have also revealed that most acupoints are situated at a nerve trunk and adjacent to or located at an artery and/or vein [31–33]. Therefore, acupuncture-stimulation-evoked NO release in the acupoint may depend on the rich distribution of neural fibers and blood vessels in the area. Our study showed that the release of NO in contralateral acupoint after LA removal significantly increased, but not on nonacupoint, which supports the previous result [16], which accords with the reporting that the NO concentration correlates with the blood perfusion of the tissue [17, 28]. It is highly possible that activating the neuronal and/or endothelial NO synthesis/release system could not only enhance the release of NO but also further benefit the blood perfusion of the acupoints.

With regard to the potential effects of LA-evoked NO release in acupoints, our studies have revealed that different-intensity LA selectively increased the NO releases just from acupoint [4]. Previous studies revealed that NO release is vital to sustain cutaneous dilation during heating or heating stress in human [5, 28, 34]. Heating on the skin can affect the endogenous NO and noradrenaline, which promote the temperature threshold of the axon reflex [35]. The majority of acupoints located on human body possess the characteristics of the low skin resistance and high electrical conductance [36, 37]. In addition, human studies have shown that acupuncture stimulation can elevate the NO level in the blood and acupuncture-stimulation-induced local circulation is closely related to NO increase in treated regions [8]. In acupuncture clinical practices, due to acupuncture works locally, herein, treating the soft-tissue damage and pain-related syndromes could be achieved by stimulating the acupoint situated at the pressure pain site [3, 8]. Stimulation of the acupoints which are discrete sites on the body is capable of activating production through appropriate neural pathways and thereby produces central effects for the treated area [31, 38, 39]. Interestingly, the changes in both the blood circulation and NO all were different between the contralateral and ipsilateral acupoint in response to the LA treatment, which may be related to the area specificity of the LA effect. These results suggest that blood circulation and NO of the acupoints can be improved by performing LA treatments to the contralateral acupoints even if the disease is on the other side. However, the mechanism responsible for LA-induced NO increase in the treated and nontreated acupoints is still uncertain; more unresolved problems need to be come true for future study.

## 5. Conclusion

Although LA-induced NO release in the PC6 acupoints for the nontreated and treated sides all significantly increased after LA removal, the NO amounts of the treated sides are obviously higher than those of nontreated sides. There are no significant changes for the NO amount in nontreated acupoints during LA stimulation; in addition, LA at RPC6 induced more NO release in bilateral PC6 than stimulating LPC6, but these phenomena did not occur at nonacupoints. The results suggested that the increases of LA-induced NO release for the contralateral acupoint and the different levels of NO release resulting from the lateralized specificity all are specific to the acupoint. LA-evoked NO release in acupoints could contribute to the blood circulation and further pain relief via local vasodilation and other possible effects.

## Figures and Tables

**Table 1:** The changes in NO_2_^−^ absorbance at RPC6 and LPC6 acupoints during LA treatment and recovery period (mean ± SE).

Group	NO release at air	NO release at RPC6	NO release at LPC6
NO_2_^−^ absorbance from the air and skin acupoint area (a.u.)	0.32 ± 0.03	0.46 ± 0.04	0.44 ± 0.04
The real NO_2_^−^ absorbance from skin acupoint area (a.u.)	0	0.14 ± 0.01^*∗*^	0.12 ± 0.02^*∗*^
LA-induced NO_2_^−^ absorbance during LA at CPC6 (a.u.)	0	0.03 ± 0.01	0.12 ± 0.01^#^
LA-induced NO_2_^−^ absorbance after LA at CPC6 (a.u.)	0	0.18 ± 0.02^&^	0.26 ± 0.03^#&^
LA-induced NO_2_^−^ absorbance after LA at itself (a.u.)	0	0.53 ± 0.05^#&$^	0.40 ± 0.04^&$^

^*∗*^ *P* < 0.05, versus at air; ^#^*P* < 0.05, LA at RPC6 versus LA at LPC6; ^&^*P* < 0.05, versus the real LA-induced NO_2_^−^ absorbance during LA at CPC6; ^$^*P* < 0.05, versus the real LA-induced NO_2_^−^ absorbance after LA at CPC6; SE, standard error.

**Table 2:** The changes of NO_2_^−^ absorbance at right and left nonacupoints during LA treatment and recovery period (mean ± SE).

Group	NO release at air	NO release at Rnon-acupoint	NO release at Lnon-acupoint
NO_2_^−^ absorbance from the air and skin nonacupoint area (a.u.)	0.32 ± 0.03	0.39 ± 0.05	0.36 ± 0.04
The real NO_2_^−^ absorbance from skin nonacupoint area (a.u.)	0	0.07 ± 0.02^*∗*^	0.04 ± 0.01^*∗*^
LA-induced NO_2_^−^ absorbance during LA at Cnon-acupoint (a.u.)	0	−0.02 ± 0.01	0
LA-induced NO_2_^−^ absorbance after LA at Cnon-acupoint (a.u.)	0	−0.02 ± 0.01	0
LA-induced NO_2_^−^ absorbance after LA at itself (a.u.)	0	0.16 ± 0.02^&^	0.22 ± 0.03^&^

^*∗*^ *P* < 0.05, versus at air; ^&^*P* < 0.05, versus the real LA-induced NO_2_^−^ absorbance during and after LA at Cnon-acupoint; SE, standard error.

## Abbreviations

TCM: Traditional Chinese Medicine
LA: Laser acupuncture
PC: Pericardium
PC6: Neiguan acupoint
RPC6: Right PC6
LPC6: Left PC6
CPC6: Contralateral PC6
Rnon-acupoint: Right noacupoint
Lnon-acupoint: Left nonacupoint
Cnon-acupoint: Contralateral nonacupoint
NO: Nitric oxide
NO_2_^−^: Nitrite
NO_3_^−^: Nitrate
NO_*x*_^−^: NO metabolites
PTIO: 2-Phenyl-4,4,5,5-tetramethylimidazoline-1-oxyl 3-oxide
NO_*x*_^−^: (NO_2_^−^, NO_3_^−^)
NOS: Nitric oxide synthase.
